# Controversies in the management of arthrocentesis treatment of temporomandibular joint disorders: systematic review

**DOI:** 10.25122/jml-2024-0402

**Published:** 2025-08

**Authors:** Tubanza Mulongo Simplot, Nyimi Bushabu Fidele, Mantshumba Milolo Augustin, Paka Lubamba Grace, Muyembi Mwinaminayi Pierre, Zinio Mabanza Julia, Panoumvita Kapamona Junior

**Affiliations:** 1 Department of Oral and Maxillofacial Surgery, Oral Maxillofacial-Head and Neck Oncology Service, Faculty of Dental Medicine, University of Kinshasa, Kinshasa, Democratic Republic of Congo; 2 National Center Laboratory of Oral Biomedicine, Ministry of Research, Innovation and Technology, Democratic Republic of Congo; 3 Department of Dental and Maxillofacial Prosthesis, Faculty of Dental Medicine, University of Kinshasa, Kinshasa, Democratic Republic of Congo; 4 Department of Oral and Maxillofacial Surgery-Head and Neck Oncology, West China Hospital of Stomatology, Sichuan University, Sichuan, China

**Keywords:** arthrocentesis, adjunctive therapy, temporomandibular joint, treatment outcome

## Abstract

There is a paucity of evidence regarding whether the effective outcomes of arthrocentesis in the management of temporomandibular joint disorders (TMJD) result from its use as a single treatment or in association with adjunctive therapy. The study aimed to compare arthrocentesis alone (ACA) and arthrocentesis (AC) associated with adjunctive therapy (AAAT) to determine the most effective treatment strategy. A systematic review was conducted in September 2023 using PubMed/MEDLINE, Scopus, Web of Science, and the Cochrane Oral Health database. Outcomes assessed included pain, maximal interincisal opening (MIO)/maximum mouth opening (MMO), and joint sounds. A total of 28 studies comprising 1,216 patients treated with ACA or AAAT were included. Across these studies, 31 temporomandibular disorder diagnoses were reported, with temporomandibular joint osteoarthritis (TMJ-OA) being the most common (32.3%), followed by temporomandibular joint internal derangement (TMJ-ID) (19.4%) and disc displacement without reduction (DDwoR) (12.9%). Comparisons showed no significant differences between ACA and arthrocentesis combined with hyaluronic acid (AC + HA) or corticosteroids (AC + CS) in most studies. Arthrocentesis combined with platelet-rich plasma (AC + PRP) was superior to ACA but generally less effective than arthrocentesis combined with PRP and HA (AC + PRP + HA). The benefit of adjunctive therapy after AC remains controversial; however, an additional effect of two different adjunctive modalities after AC seems evident.

## INTRODUCTION

Temporomandibular disorders (TMD) are musculoskeletal conditions characterized by facial pain and impaired temporomandibular joint (TMJ) function [1]. TMD can be acute or chronic and is ranked the third most common oral and maxillofacial disease after dental caries and periodontal diseases, with an estimated incidence of 12% per year [2-4]. The most common signs and symptoms are facial pain, limited mouth opening, and TMJ sounds during movements, and all these signs may result in a severely impaired quality of life (QOL) [5]. Although the treatment of TMD remains controversial, two types of treatment strategies were described and represent the most reported treatments for TMD in the literature, including conservative management and surgical intervention [6,7].

Conservative management modalities include physical therapy, pharmacotherapy, lifestyle adaptations, and occlusal appliance splint therapy. Surgical management can be classified into invasive open methods and minimally invasive procedures such as arthrocentesis (AC), intra-articular steroid injection, and arthroscopy [8]. TMJ arthrocentesis is a minimally invasive surgical procedure indicated for patients with internal joint derangements or other inflammatory arthropathies unresponsive to nonsurgical management. It is currently one of the most widely used and popular treatment options for TMD, second only to arthroscopy [2,9]. The primary objectives of arthrocentesis are to irrigate the joint to remove inflammatory mediators, release the articular disc, break intra-articular adhesions, alleviate pain, and improve joint mobility. This technique is technically simple, feasible without the need for complex instrumentation, associated with a low complication rate, and effective in improving TMJ osteoarthritis (OA), restoring joint function from a dysfunctional to a functional state [10,11].

However, it remains unclear whether the satisfactory outcomes of arthrocentesis (AC) in the management of temporomandibular joint disorders (TMJD) are attributable to its use as a standalone treatment or in combination with adjunctive therapies (AT). Several adjunctive treatments have been combined with AC in an attempt to enhance its therapeutic effect, including AC with hyaluronic acid (AC + HA), platelet-rich plasma (AC + PRP), glucosamine, chondroitin sulfate, methylsulfonylmethane (GCM), platelet-rich fibrin (AC + PRF), and corticosteroids [12-15]. Some studies suggest that adjunctive therapies may contribute to the good outcome observed after AC. However, such studies are few and controversial; therefore, their outcomes should be interpreted cautiously. The highest level of response for this issue may be gained from a systematic review, which allows for collecting and synthesizing relevant primary data on a particular interest [16,17]. Therefore, the present study aimed to compare arthrocentesis alone (ACA) with arthrocentesis combined with adjunctive therapies (AAAT) to determine the most effective treatment strategy and identify specific indications based on the available evidence.

## MATERIAL AND METHODS

This study followed the PRISMA (Preferred Reporting Items for Systematic Reviews and Meta-Analyses) guidelines [18]. The research question was structured using the PICO framework: in patients with temporomandibular joint disorders (TMJD) (P), does temporomandibular joint (TMJ) arthrocentesis alone (I), compared with arthrocentesis combined with adjunctive therapy or other control treatments (C), provide better clinical outcomes (O)?

### Protocol and registration

The protocol for this study was registered with the International Prospective Register of Systematic Reviews (PROSPERO)—Registration number: CRD42023477330.

### Search strategies

An electronic search was conducted in September 2023 across PubMed/MEDLINE, Scopus, Web of Science, and the Cochrane Oral Health database without time restrictions but limited to English-language publications. Both Medical Subject Headings (MeSH) terms and free-text keywords were used, including: (temporomandibular arthrocentesis) OR (TMJ AND arthrocentesis) OR (Treatment of TMJD) AND (arthrocentesis) OR (arthrocentesis treatment). The reference list of the identified studies and relevant reviews on the subject was also screened for possible additional studies.

### Eligibility criteria and types of studies

Eligibility criteria included peer-reviewed studies limited to clinical series, randomized controlled trials (RCTs), cohort studies, case-control studies, cross-sectional studies, retrospective comparative studies, and case series of TMJD written in English, reporting the use of arthrocentesis alone or associated with adjunctive therapy. Studies were required to clearly define the evaluated variables and, where possible, provide diagnoses based on the Diagnostic Criteria for Temporomandibular Disorders (DC/TMD) or the Research Diagnostic Criteria for Temporomandibular Disorders (RDC/TMD) [19]. Additionally, only articles available as full texts that presented the descriptors in their title, abstract, or main text were included. Studies reporting less than 5 cases of TMJD, immunohistochemically studies, epidemiological studies, radiological studies, genetic expression studies, histopathological studies, cytological studies, in vitro studies, studies that included the agenesis, hyperplasia, hypoplasia, bone ankyloses cases, previous TMJ surgery, conservative treatment methods (physiotherapy, splint therapy, and pharmacotherapy), invasive surgical procedures (open joint surgery) and review papers were excluded.

### Population

The study population comprised subjects diagnosed with TMJD according to RDC/TMD or DC/TMD criteria in the included studies.

### Comparator

Eligible studies compared outcomes of TMJ arthrocentesis performed alone (ACA) versus arthrocentesis combined with adjunctive therapy (AAAT).

### Outcomes

The primary outcome was to identify the most effective adjunctive therapy when combined with arthrocentesis for TMJD. The secondary outcome was to determine the most frequent indications for the selected surgical approach.

### Study selection

In the first phase, three recalibrated authors independently read and evaluated the titles and abstracts of the papers identified in the electronic databases. Studies meeting inclusion criteria, or those with insufficient data in the title and abstract, were retrieved for full-text review. Two authors independently reviewed the full texts, and disagreements were resolved by consensus or consultation with a third, more experienced author. In the second phase, when the papers were read in full, the opinion of a third author was again requested when the two authors disagreed and did not reach a consensus for the final inclusion of the selected studies.

### Data collection process and data items

A data collection sheet was completed for each article that met the inclusion criteria. Data were extracted independently by the same three authors using standardized data collection sheets for final analysis. Sample size, demographic data, treatment modality, type of adjunctive treatment, previous TMJ treatment, raising fluid and volume, follow-up time, technical type of AC, type of anesthesia, and the measurement variables for performing arthrocentesis alone or with adjunctive therapy were all recorded.

### Risk of bias

The selected studies were assessed for level of evidence as per the Oxford Center for Evidence-Based Medicine (CEBM) guidelines, which classifies clinical studies from level 1 to 5, with level 1 being the highest (randomized control trials), level 2 (prospective cohort studies), level 3 (retrospective case-controlled studies), level 4 (case-series), and finally level 5 (case-based reasoning and laboratory studies). The same authors performed the assessment.

### Strategy for data synthesis

After verifying the availability of articles, the titles and abstracts of all the records obtained from the literature search were screened, and the full texts of the records meeting the inclusion criteria were retrieved for further evaluation. After the screening, the bibliography of the included studies and review articles on the subject was hand-searched to ensure that no important references were missed. All the reported outcomes and methods were identified, and they were recorded in a standardized data extraction sheet designed in Microsoft Excel with information about the authors, year of publication, study design, TMJ pathological condition requiring treatment (a TMD diagnosis), sample size, number of case subjects, technique used, drug injected, number of control subjects, and the main results (post-therapeutic outcomes). Data were assembled into an evidence table, and a detailed summary was performed to ascertain the quality of the data and the level of the study. The objective was to identify the clinical indication and compare other associated treatments that can provide a good outcome. The study protocol was reviewed and approved by the Institutional Review Board of our institution.

## RESULTS

**Figure 1:**
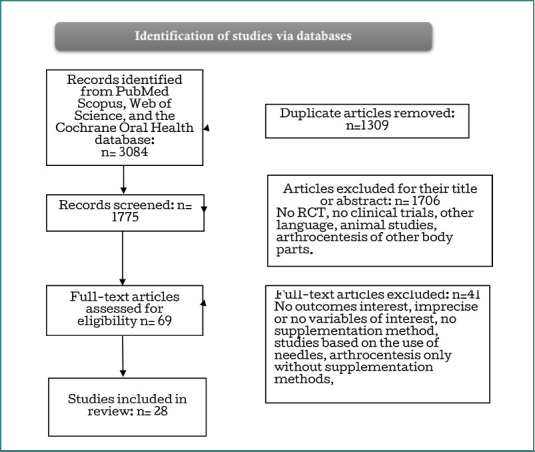
PRISMA flowchart of the selected articles

A total of 3,084 items were identified through database searches, among which 1,309 articles were duplicated and removed. After independent screening of the titles and abstracts of the remaining 1,775 studies for their eligibility, a total of 1,706 studies were excluded as unrelated to the topic. The full text of the remaining 69 articles was reviewed for the assessment of inclusion criteria, which led to the exclusion of 41 that did not meet the inclusion criteria. Finally, 28 studies on the management of TMJD (or TMD) arthrocentesis alone or associated with adjunctive therapy [12-15,20-24] were included in this review for further analysis. The flowchart of the study selection process is summarized in Figure 1.

Across all included studies, a total of 1,216 patients were treated using either arthrocentesis alone or arthrocentesis combined with adjunctive therapy (Table 1). Sample sizes ranged from 20 to 120 patients per study. Most studies were published in 2021 and 2023, followed by 2016 and 2017. Fourteen out of twenty-eight studies were conducted in Turkey, and 27 studies were RCTs, classified as level 1 according to the CEBM guidelines. The study variables most reported were pain (25 out of 28 studies), maximal interincisal opening (MIO)/maximum mouth opening (MMO) (24 out of 28 studies), and joint sounds (10 out of 28 studies). The follow-up time ranged from three to 48 months. Among the 31 temporomandibular disorder (TMD) diagnoses reported across these 28 studies, temporomandibular joint osteoarthritis (TMJ-OA) was the most common (32.3%), followed by temporomandibular joint internal derangement (TMJ-ID) (19.4%) and disc displacement without reduction (DDwoR) (12.9%) (Table 1).

**Table 1:** Characteristics of the included studies using arthrocentesis alone or associated with adjunctive therapies

Author	Year	Country	Design	Indications	Sample size	Variables	Conclusions
Hegab *et al*. [12]	2023	Egypt	RCT	TMJ-OA	90	Pain MMO Joint sounds	PRP+HA showed statistically significant improvement of the MIO, pain, and joint sound outcomes compared to PRP or HA injection.
Karadayi *et al*. [13]	2021	Turkey	RCT	TMJ-ID	36	Pain MIO HCDS	The AC + i-PRF gave much better outcomes than AC alone
Kiliç *et al*. [14]	2021	Turkey	RCT	TMJ-OA	26	Pain, MIO:	The use of GCM after AC+ HA injection produced no additional benefits.
Huddleston Slater *et al*. [15]	2012	Netherlands	RCT	TMJ Arthralgia	28	Pain MIO	IAI Dexamethasone following AC did not improve the procedure’s effect in patients with TMJ Arthralgia
Hassan *et al*. [20]	2023	Iraq	CRT	TMJ-ID	60	Pain Joint sounds MMO	More favorable treatment outcomes for ozonized water lavage, and it is a promising new treatment modality for the relief of symptoms associated with the TMJ-ID.
Bayramoglu *et al*. [21]	2023	Turkey	RCT	TMJ-OA	30	Pain MMO Joint sounds	AC+ Tenoxicam showed no better outcomes in terms of Pain, MMO, and joint sounds compared with Ac alone.
Işık *et al*. [22]	2022	Turkey	RCT	TMJ-OA	36	Pain MMO	IAI of i-PRF after AC should be preferred.
Dasukil *et al*. [23]	2022	India	RCT	TMJ-ID	90	MIO: Pain: Joint sounds	PRP may be preferable to HA following AC.
Sembronio *et al*. [24]	2021	Italy	RCT	TMJ-ID, TMJ-OA	40	Pain MIO	Micro-fragmented adipose tissue can significantly improve outcomes of pain and function compared with the standard treatment
Dolwick *et al*. [25]	2020	USA	RCT	N/S	24	Pain MMO	Support steroid supplementation after TMJ AC to increase pain-free mandibular mobility.
Bergstrand *et al*. [26]	2019	Norway	RCT	TMJ-OA	37	Pain MIO Joint sounds	Both methods resulted in significant long-term improvement in pain and jaw function
Yapici-Yavuz *et al*. [27]	2018	Turkey	RCT	DDwoR	44	N/S	AC alone, AC with Methylprednisolone acetate or HA, or Tenoxicam IAI are similarly effective and promising methods in the treatment of TMJ-DDwoR.
Bouloux *et al*. [28]	2017	USA	RCT	-Arthralgia, -Disc displacement, -DJD	98	Pain	ACA is as efficacious as AC with HA or CS in reducing TMJ pain
Tatli *et al*. [29]	2017	Turkey	RCT	DDwoR	120	Pain Jaw function Disability and psychological status	Splint application has no additional effect on the effectiveness of AC
Ozdamar *et al*. [30]	2017	Turkey	RCT	TMJ-ID	24	Pain MIO	AC improves both pain and MIO scores over time, but these parameters do not differ between patients receiving either AC alone or AC+HA.
Kiliç *et al*. [31]	2016	Turkey	RCT	TMJ-OA	31	Pain MIO	AC+ PRP injection is not superior to AC+ HA injection.
Kiliç *et al*. [32]	2016	Turkey	RCT	TMJ-OA	24	Pain Joint sound MIO	AC+CS produced no better outcomes compared to AC alone.
Patel *et al*. [33]	2016	India	RCT	TMJ-ID	30	Pain MIO	AC + HA injection seemed to be superior to AC alone
Kiliç *et al*. [34]	2015	Turkey	RCT	TMJ-OA	30	Pain Joint sounds MIO:	AC+PRP injections constitute a safe and promising method for the treatment of TMJ OA that is superior to AC alone.
Sipahi *et al*. [35]	2015	Turkey	RCT	TMJ-ID	30	Pain MMO	Morphine given by IAI after AC gives a significant, sustained improvement in pain relief compared with simple AC alone. The effect was similar to Tramadol except that it was shorter-lived.
Tabriz *et al*. [36]	2014	Iran	RTC	TMJ-ID	60	Pain MMO Joint sounds	AC using LRS with or without corticosteroids may have the same effect on pain relief.
Olsen-Bergem *et al*. [37]	2014	Norway	RCT	JIA	21	Pain MIO PIO	Steroids had no additional effect
Ghanem *et al*. [38]	2011	Egypt	Comparative study	ICLB	20	Pain: MIO:	AC and stabilizing splints are the treatment of choice for an acute closed lock of short duration in young patients with bruxism.
Machon *et al*. [39]	2011	Czech Republic	RCT	TMJ-OA	80	N/S	AC+ splint is an effective first-stage treatment method for patients with TMJ OA.
Aktas *et al*. [40]	2010	Turkey	RCT	DDwoR	21	Pain MMO Joint sounds	Both treatments successfully increased MMO and pain.
Alpaslan et all. [41]	2008	Turkey	RCT	DDwoR	25	Pain MMO	The use of splints as an additional therapy does not affect the short-term prognosis.
Alpaslan *et al*. [43]	2001	Turkey	RTC	DDwR	31	Pain MMO Clicking sounds	AC+HA seemed to be superior to AC alone.
Harba *et al*. [42]	2022	Syria	RCT	N/S	24	Pain Joint sounds Minimum IO Bite force	AC + PRP was found to be more effective than AC alone

GCM, Glucosamine, Chondroitin sulfate, and Methylsulfonylmethane; AC, Arthrocentesis; ACA, Arthrocentesis alone; IAI, Intra-Articular Injection; HA, Hyaluronic Acid; RCT, Randomized Controlled Trial; TMJ-OA, Temporo-Mandibular Osteo Arthritis; MIO, Maximal Interincisal Opening; PRP, Platelet-Rich Plasma; TMJ ID, TMJ Internal Derangement; i-PRF, Injectable platelet-rich fibrin; CS, Corticosteroid; DDwoR, Disc displacement without reduction; DDwR, Disc displacement with reduction; MMO, Maximum mouth opening; LRS, Lactated Ringer Solution. N/S, not specified; ICLB, Intermittent closed lock with bruxism; IO, Interincisal opening; HCDS, Helkimo clinical dysfunction score; PIO, Pain Incisal Opening

Among the adjunctive methods evaluated in association with AC (Table 2), hyaluronic acid (HA) was the most frequently used (10/28 studies), followed by corticosteroids (CS: dexamethasone, triamcinolone, methylprednisolone) (6/28 studies), platelet-rich plasma (PRP) (6/28 studies), and splint therapy (5/28 studies). Most studies assessing AC combined with HA (4/11) reported no significant difference in outcomes compared with arthrocentesis alone (ACA), similar to findings for AC combined with corticosteroids. AC combined with PRP demonstrated superior outcomes compared with ACA but was generally less effective than AC combined with PRP and HA (AC + PRP + HA) in most studies.

**Table 2:** Treatment methods of arthrocentesis and outcome

Author	Gender	Mean age	Diagnosis	Concentration solution	Number of interventions and concentration of adjuvants	Type of AC Joint space	Diameter (Gauge)	Follow-up	Anesthesia type	Benefit effect
Alpaslan *et al*. [41]	41 females 4 males	30.1 11	DDwoR	RL 100 cc	-AC alone -AC+ hard splints -AC+ soft splints	2 needles Upper space	21-gauge	6	LA	None: Splint therapy
Kiliç *et al*. [32]	21 females 3 males	33.84 12.21	TMJ-OA	RL 100 cc	-1 AC alone -1 IAI of 1 ml of MPA (CS) after 1 AC	2 needles N/S	20-gauge	16	LA	None: MPA (CS)
Bouloux *et al*. [28]	N/S	45.2	-Arthralgia -Disc displacement -DJD	RL 200 cc	-1 IAI of 1 ml of HA after 1 AC -1 IAI of 1ml of CS after 1 AC -1 IAI of 1 ml of RL after 1 AC	2 needles Upper space	21-gauge	3	LA or LA+ IV Sedation	None: HA (Hyalgan) CS (Celestene)
Bergstrand *et al*. [26]	30 females 7 males	51	TMJ-OA	RL	-1 AC alone -1 IAI of 1 ml of HA after 1 AC	2 needles N/S	N/S	48	LA	None: HA (Synvisc)
Aktas *et al*. [40]	17 females 4 males	26.43	DDwoR	N/S	-1 AC alone -1 IAI of 1 ml of Tenoxicam after 1 AC + Stabilization splint for both groups	2 needles Upper space	19-gauge	6	LA	None: Tenoxicam + Splint therapy
Alpaslan *et al*. [43]	26 females 5 males	27	DDwR	SS 200 to 300 cc	-1 AC alone -1 IAI of 1 ml of HA after 1 AC	2 needles Upper space	20-gauge	24	LA	Yes: HA (Orthovisc)
Bayramoglu *et al*. [21]	24 females 6 males	41.96 ± 11.50	TMJ-OA	RL 100 cc	-1 AC alone -1 IAI of 2 ml of Tenoxicam after 1 AC	2 needles N/S	N/S	6	LA	None: Tenoxicam
Dolwick *et al*. [25]	22 females	48.9 5.6	TMJ Pain	RL 100 cc	-1 AC -1 IAI of 1 ml of combination of 2.5 mg bupivacaïne and 20 mg Triamcilone (CS)	2 needles Upper space	19-gauge	4	AG	Yes: Bupivacaïne + Triamcilone (CS)
Sipahi *et al*. [35]	25 females 5 males	Between 16 and 50 years	TMJ-ID	RL 60 – 100 cc	1 IAI of 1 ml of RL after 1 1AC 1 IAI of 0.01 g of Morphine after 1 AC 1 IAI of 50 mg of Tramadol after 1 AC	2 needles Upper space	20-gauge	6	LA	Yes: Opioids
Tabriz *et al*. [36]	47 females 13 males	27.85 ± 7.30	TMJ-ID	RL 200 cc	1 AC alone 1 IAI of 8 mg of Dexamethasone (CS) after 1 AC	2 needles Upper space	21-gauge	6	LA	None: CS
Tatli *et al*. [29]	107 females 13 males	28.75 ± 11.30	DDwoR	IS 120 cc	1 IAI of 2 ml of HA after 1 AC 1 IAI of HA after 1 AC+ Splint therapy Splint therapy only	2 needles Upper space	21-gauge	6	LA	None: Splint therapy
Hassan *et al*. [20]	45 females 15 males	Between 14 and 66 years	TMJ-ID	OW RL	1 AC with OW as irrigation solution 1 AC with RL as irrigation solution	2 needles N/S	21-gauge	4	LA	Yes: OW
Isık *et al*. [22]	33 females 3 males	45.20 ± 12.6	TMJ-OA	SS 200 cc	-1 IAI of 1 ml of i-PRF after AC + 4 sessions of 1 ml of i-PRF without AC - 1 AC alone	2 needles Upper space	20-gauge	12	LA	Yes: i-PRF
Dasukil et all. [23]	64 females 26 males	37.4 ± 4.9	DDwR	RL 100 cc	1 AC+ 1 IAI of 3 – 4 ml of RL 2 IAI of 1 ml of HA after AC 2 IAI of 1 ml of PRP after AC	1 needle N/S	21-gauge	6	LA	Yes: PRP
Harba *et al*. [42]	N/S	27.25	N/S	N/S	4 IAI of 1 ml of HA after 4 AC 4 IAI of 0.5 ml of HA & 0.5 ml of PRP after 4 AC	N/S Upper space	19-gauge	6	N/S	Yes: PRP+HA (Hyalgan®)
Kiliç *et al*. [34]	27 females 3 males	33.65 14.58	TMJ-OA	RL 100 cc	-1 AC alone -1 IAI of 1 ml of PRP after AC + 4 sessions of 1 ml of PRP injection without AC	2 needles N/S	20-gauge	12	LA	Yes: PRP
Kiliç *et al*. [14]	23 females 3 males	28.35 ± 10.85	TMJ-OA	RL 100 cc	-1 IAI of 2 ml of HA after 1 AC -1 IAI of 2 ml HA after 1 AC, followed by 3 months of oral GCM	2 needles N/S	20-gauge	12	LA	None: GCM
Kiliç *et al*. [31]	26 females 5 males	30.48 13.04	TMJ-OA	RL 100 cc	-1 IAI of 1 ml of PRP after AC + 4 sessions of 1 ml of PRP injection without AC -1 IAI of 1 ml of HA after 1 AC	2 needles N/S	20-gauge	12	LA	None: PRP HA (Hyalgan®)
Yapici-Yavuz *et al*. [27]	38 females 6 males	N/S	DDwoR	RL 200 cc	- ACA - AC+ MPA (CS) - AC+ HA - AC+ Tenoxicam	2 needles N/S	N/S	6	LA	None: CS HA (Hyalgan®) Tenoxicam
Hegab *et al*. [12]	58 females 32 males	31.5 5.2	TMJ-OA	SS 200 cc	-1 IAI of 2 ml of PRP after 1 AC -1 IAI of 2 ml of HA after 1 AC -1 IAI of 2 ml of PRP mixed with HA after 1 AC	2 needles N/S	19-gauge	12	GA	Yes: PRP+HA
Ghanem *et al*. [38]	20 females	34	ICL with bruxism	RL 200 cc	1 AC + hard splint 1 AC Every group received 1 ml of Betamethasone	2 needles Upper space	19-gauge	12	LA	Yes: Splint therapy
Huddleston Slater *et al*. [15]	23 females 5 males	33.3	Arthralgia	IS 300 cc	- 1 ml of IS after 1 AC - 1 IAI of 1 ml of Dexamethasone (CS) after 1 AC	2 needles Upper space	18-gauge	6	LA	None: CS
Karadayi *et al*. [13]	19 females 17 males	39.82	TMJ-ID	RL 100 cc	- 1 IAI of 2 ml of i-PRF after 1 AC - 1 AC alone	2 needles N/S	20-gauge	3	LA	Yes: i-PRF
Machon *et al*. [39]	61 females 19 males	52.8	TMJ-OA	RL 120 cc	- Rest therapy - Splint therapy - 1 AC+ 2 ml of 1 IAI of HA - 1 AC+ 2 ml of 1 IAI of HA+ Splint therapy	2 needles Upper space	N/S	3	LA	Yes: Splint therapy
Olen-Bergem *et al*. [37]	15 females 6 males	11.4	JIA, TMJ-Arthritis	SS 140 cc	- 1 AC alone -1 IAI of Triamcilone (CS) after 1 AC	N/S N/S	N/S	8	GA	None: CS
Ozdamar *et al*. [30]	17 females 7 males	26.87 ± 7.92	TMJ-ID	N/S	- 1 IAI of 2 ml of HA after AC (2 sessions) - 1 IAI of 2 ml of SS after 1 AC (2 sessions)	2 needles Upper space	20-gauge	3	N/S	None: HA (Orthovisc®)
Patel *et al*. [33]	21 females 9 males	N/S	TMJ-ID	RL 200 – 300 cc	- 1 AC alone - 1 IAI of 1 ml of HA after 1 AC	2 needles Upper space	18-gauge	6	LA	Yes: HA
Sembronio *et al*. [24]	31 females 9 males	43.5	TMJ-ID, TMJ-OA	SS 200 cc	- 1 IAI of 2 ml of HA after 1 AC - 1 IAI of adipose Tissue after 1 AC	2 needles Upper space	19-gauge	6	LA	Yes: Adipose Tissue

RL, Ringer’s lactate; SS, Saline solution; MRI, Magnetic resonance image; LA, Local anesthesia; GA, General anesthesia; MPA, methylprednisolone acetate; DJD, Degenerative joint disease; OPG, Orthopantomogram; CT, Computed tomography; IV, intravenous; IAI, Intra articular injection; 1 AC, One session of Arthrocentesis; CS, Corticosteroid; ICL, Intermittent closed lock; IS, Isotonic saline; ITMD, internal temporomandibular disorder; N/S, Not specified; ID, Internal Derangement; AAOMS, American Association of Oral and Maxillofacial Surgery; OZ, Ozonized water.

Of the 28 randomized controlled trials (RCTs) [12-15, 20-22, 24-37, 37-43] included in this review, 25 used the two-needle technique (2NT) [12-15,20-22, 24-36,38-41,43], one used the one-needle technique (1NT) [23], and two did not specify the technique used [37,42]. Sixteen RCTs reported inserting needles into the upper joint space [15,22,24,25,28-30,33,35,36,38-43]. Regarding needle gauge, 20-gauge needles were the most commonly used (9 RCTs) [13,14,22,30-34,35,43], followed by 21-gauge and 19-gauge needles, each used in six RCTs [20,23,28,29,36,41] and [12,24,25,38,40,42]. Only two RCTs reported using 18-gauge needles [15,33], while five studies did not specify the gauge [21,26,27,37,39]. The benefit of AAAT was not associated with patient sex, number of interventions, or concentration of adjuvant.

Eight RCTs evaluated the effectiveness of intra-articular injection (IAI) of hyaluronic acid (HA) following AC [23,26-28,30,31,33,43]; six of these reported no additive effect from this combination. In contrast, in six RCTs comparing ACA with AC coupled with CS [16,25,27,28,36,37], only one study found a beneficial effect of CS after AC, specifically for triamcinolone combined with bupivacaine. When comparing ACA vs AC+PRF or PRF+HA in five studies [13,23,31,34,43], four found a beneficial effect of PRP as an adjunctive therapy after AC. Regarding splint therapy, five RCTs investigated its effectiveness when combined with AC [29,38,39,40,41]. Only two reported a positive effect, one of which specifically involved patients with bruxism [38]. Two RCTs [14,21] evaluated the efficacy of AC followed by intra-articular injection of injectable platelet-rich fibrin (i-PRF) vs ACA, both of which demonstrated improved outcomes compared with ACA. One RCT investigated the use of opioids (morphine and tramadol) as adjunctive agents following AC [35], reporting beneficial effects in 30 patients with temporomandibular joint internal derangement over a 6-month follow-up. Additionally, three RCTs compared ACA vs AC+ tenoxicam [20,27,40], and one RCT evaluated intra-articular injection (IAI) of glucosamine/chondroitin/methylsulfonylmethane (GCM) following AC + HA [15]; none of these studies reported any additional benefit. In contrast, one RCT found that using ozonized water as the irrigating fluid for AC resulted in improved outcomes compared with Ringer’s lactate (RL). Across the 28 RCTs, the most commonly reported irrigating solution was RL [13,14, 20-23, 25-27,32-36, 38-41], followed by saline solution (SS) [12,22,24,37,43], isotonic serum (IS) [15,29], and ozonized water [20]. The volume of RL used ranged from 60 to 200 mL, while SS volumes ranged from 140 to 300 mL. The number of AC sessions varied from 1 to 4, with adjunctive IAI sessions also ranging from 1 to 4, sometimes administered in combination with a single AC procedure.

## DISCUSSION

The effective management of arthrocentesis treatment of temporomandibular joint disorders as a single procedure or associated with adjunctive therapy is still challenging. Several adjunctive therapies associated with arthrocentesis have been described in the literature, and the findings appear to suggest that these factors may predict a good outcome. The present study aimed to compare the arthrocentesis treatment alone and arthrocentesis associated with adjunctive therapy to determine the most effective treatment methods and their indications based on available evidence. Some studies revealed that until 2019, almost 191 systematic reviews on TMD have been published and focused on the evaluation of the most effective management strategies for various TMD types [16,17]. The authors concluded that there was moderate evidence to support a multi-modal conservative approach towards the initial management of TMD. However, the use of arthrocentesis or arthroscopy has shown benefits in cases of heterogeneous TMD where conservative measures fail.

From 2017 to 2023, hyaluronic acid was the most commonly reported injectable administered into the temporomandibular joint cavity [44]. Several authors have confirmed its short-term effectiveness in reducing joint and muscle pain in patients with articular disc displacement [45]. However, Ferreira *et al*. [46], in a systematic review, found no superiority of arthrocentesis combined with HA (AC + HA) compared with arthrocentesis alone (ACA). In the present review, out of eight RCTs evaluating the effectiveness of intra-articular HA following AC [23,26-28,30-33,43], six reported no additional benefit from this combination. Corticosteroid (CS) injections are widely used for TMJ pain management and have been shown to reduce pain [47] effectively. For instance, the use of intra-articular methylprednisolone has been reported to improve mouth opening capacity within approximately 3 weeks [48]. Additionally, a study comparing the efficacy of triamcinolone alone as an intra-articular corticosteroid injection and ACA found that both techniques were successful in reducing pain and improving mouth opening, in both the short and long term. These findings contrast with those of the current review, in which six RCTs [16,25,27,28,36,37] reported no added benefit of CS injections following AC. Similarly, other authors have observed that ACA alone produced superior long-term outcomes compared with CS injections alone in managing TMJ internal derangement (case of triamcinolone used in their study) [49]. Liu *et al*. [50] compared combined AC with intra-articular CS injections versus ACA alone and reported no significant differences between groups in terms of pain intensity and MMO in the short term; however, significant improvement was observed in the CS group at long-term follow-up. The present review found no evidence to support an additive effect of CS coupled with AC compared to ACA. Based on this, we suggest the use of CS as an adjuvant to AC in cases where CS is indicated.

A previous study reported that TMD treated with PRP injection had slightly better outcomes [51]. These intra-articular injections of PRP were more effective in reducing the symptoms than ACA (using normal saline). In the present review, five RCTs comparing arthrocentesis alone with arthrocentesis combined with PRP or PRP mixed with HA [13,23,31,34,43] reported a beneficial effect of PRP as an adjunctive modality. Our review confirms the finding of Li *et al*. [52], who, in their systematic review and meta-analysis, evaluated and compared the effectiveness of diverse therapies for disc displacement of TMJ, such as AC, injections with diverse drugs, occlusal appliances, and splints. The authors concluded that AC+PRP provided superior outcomes in both mouth-opening improvement and pain alleviation due to the anti-inflammatory, analgesic, and lubricating effects of PRP. Regarding the splint therapy used as an adjunctive modality for improved outcomes after AC [29,38,39,40,41], no beneficial effect was found, contrary to some authors indicating that splint therapy may improve outcomes after AC [53].

The contribution of injectable platelet-rich fibrin (i-PRF) [14,21] after AC was evident in improving outcomes for the treatment of arthrogenic TMD, with follow-up periods ranging from 3 to 12 months. Sielski *et al*. [54] reported that combined therapy (AC + i-PRF) achieved superior results compared with AC alone, with a 16% to 43% greater reduction in articular pain and a 6% to 36% improvement in mandibular abduction. In addition, opioids have long been used in dentistry for managing chronic moderate-to-severe pain when conventional analgesics are insufficient. Both oral and parenteral routes have been employed, including intra-articular administration in combination with AC. In a randomized double-blind study, intra-articular morphine significantly increased the pain threshold for TMJ disease [55]. In the present review, one RCT investigated the use of intra-articular morphine and tramadol as adjuncts to AC [35], reporting beneficial effects in 30 patients over a 6-month follow-up [35]. However, no additional benefit was observed in studies comparing ACA with AC combined with tenoxicam [20,27,40] or intra-articular glucosamine/chondroitin/methylsulfonylmethane (GCM) following AC + HA [15].

Twenty-five RCTs used the 2NT [12-15,20-22,24-36,38-41,43], compared to one RCT [23] which used the 1NT. Consistent with the findings of Guarda *et al*. [56] in 2012 and Mehmet *et al*. [57] in 2017, the present review did not identify a clear advantage of one technique over the other. In contrast, Cindy Azan *et al*. [58] reported that 2NT was more effective than 1NT in reducing pain and improving mouth opening. The upper joint space is described as the largest and most accessible for needle insertion [59]. In this review, more than 15 studies reported inserting needles into the upper joint space [15,22,24,25,28,29,30,33,35,36,38-43,60], while 12 RCTs [12-14,20,21,23,26,27,31,32,34,37] did not specify the injection site. Chęciński *et al*. [59] in their systematic review and meta-analysis, concluded that there was an advantage of the upper joint space over the lower joint space. The present study could not compare the advantages of injection into the upper joint space over the lower joint space since all the RCTs specified the upper level. Based on these findings and existing literature, the authors of this review support the use of the upper joint space, which appears to be associated with improved symptom relief in TMD patients treated with ACA or AAAT.

The most commonly used needle diameter was 20-gauge [13,14,22,30,31,32,34,35,43]. Other authors have reported that needle diameter can significantly influence postoperative pain levels when using the two-needle technique [61]. Ringer’s lactate was the most frequently used irrigating solution, with reported volumes ranging from 60 to 200 mL, followed by saline solution, used in five RCTs [12,22,24,37,43] at volumes ranging from 140 to 300 mL. However, Azan *et al*. [58] found that RL was more effective than SS in reducing postoperative pain. Further studies are required to provide more evidence on the effectiveness of ACA and AAAT using IS or OW as irrigation fluids. Although this systematic review presented pooled estimates from 28 studies across the literature, our study has some limitations. First, the small sample size due to restrictions related to our inclusion criteria and possible unintentional omission of some studies. Future randomized clinical trials are needed to understand better the clinical outcomes related to the different adjunctive modalities associated with AC.

## CONCLUSION

In summary, there is no evidence to support the superiority of any adjunctive therapy after TMJ AC. The use of splint therapies as an adjunctive modality for improving outcomes of AC in the management of TMD after AC may be limited in patients with parafunction. The use of i-PRF or PRP alone or associated with HA as an adjunctive modality after TMJ AC for treatment of arthrogenic TMD may be suggested. TMJ osteoarthritis, followed by internal derangement and disc displacement, were the most frequent indications for arthrocentesis across the included studies. Based on the findings of this review, the following procedural parameters are suggested: the use of Ringer’s lactate as the irrigating fluid, with a minimum volume of 100 mL; application of the two-needle technique targeting the upper joint space; and performing at least two sessions spaced by a minimum interval of seven days (one session per week), using 19- or 20-gauge needles. ACA or AAAT remains an effective technique for relieving the symptoms of TMD. Further research on opioids, adipose tissue, ozonized water, and GCM as adjunctive therapy after TMJ AC for treatment of arthrogenic TMD should be conducted.
